# Implementation and impact analysis of a transitional care pathway for patients presenting to the emergency department with cardiac-related complaints

**DOI:** 10.1186/s12913-018-3482-2

**Published:** 2018-08-30

**Authors:** Gabriel E. Soto, Elizabeth A. Huenefeldt, Masey N. Hengst, Arlo J. Reimer, Shawn K. Samuel, Steven K. Samuel, Stephen J. Utts

**Affiliations:** 1 SoutheastHEALTH, 1701 Lacey Street, Cape Girardeau, MO 63701 USA; 2 Kearny County Hospital, Lakin, KS USA; 3 Montefiore Medical Center, Bronx, NY USA; 4 CityMD, Uniondale, NY USA; 5 Austin Gastroenterology, Austin, TX USA

**Keywords:** Care coordination, Continuity of patient care, Interdisciplinary communication, Emergency department, Patient discharge, Cardiovascular services, Healthcare disparities

## Abstract

**Background:**

Cardiac-related complaints are leading drivers of Emergency Department (ED) utilization. Although a large proportion of cardiac patients can be discharged with appropriate outpatient follow-up, inadequate care coordination often leads to high revisit rates or unnecessary admissions. We evaluate the impact of implementing a structured transitional care pathway enrolling low-risk cardiac patients on ED discharges, 30-day revisits and admissions, and institutional revenues.

**Methods:**

We prospectively enrolled eligible patients presenting to a single-center Emergency Department over a 12-month period. Standardized risk measures were used to identify patients suitable for early discharge with cardiology follow-up within 5 days. The primary endpoints were rates of discharge from the ED and 30-day ED revisit and admission rates, with a secondary endpoint including 30-day returns for myocardial infarction. A cost analysis of the program’s impact on institutional revenues was performed.

**Results:**

Among patients presenting with cardiac-related complaints, rates of discharge from the ED increased from 44.4 to 56.6% (*p* < 0.0001). Enrollment in the transitional care pathway was associated with a reduced risk of cardiac-related ED revisits (RR 0.22, *p* < 0.0001), all-cause ED revisits (RR 0.30, *p* < 0.0001), and admission at second ED visit (RR 0.56, *p* = 0.0047); among enrolled patients, the 30-day rate of return with a myocardial infarction was 0.35%. No significant reductions were seen in 30-day cardiac-related and all-cause revisits in the 12-months following transitional care pathway implementation; however, there was a significant reduction in admissions at second ED visit from 45.6 to 37.7% (*p* = 0.0338). An early gender disparity in care delivery was identified in the first 120 days following program implementation that was subsequently eliminated through targeted intervention. There was an estimated decline in institutional revenue of $300 per enrolled patient, driven predominantly by a reduction in admissions.

**Conclusions:**

A structured transitional care pathway identifying low-risk cardiac patients who may be safely discharged from the ED can be effective in shifting care delivery from hospital-based to lower cost ambulatory settings without adversely impacting 30-day ED revisit rates or patient outcomes.

## Background

Cardiac-related complaints are leading drivers of ED utilization. As the second most common reason for ED visits across all age groups, chest pain represents 5.3% of all encounters [1] and accounts for 8 to 10 million visits annually [2], although ultimately less than 10% of these patients are diagnosed with an Acute Coronary Syndrome (ACS) [3–5]. Congestive Heart Failure (CHF) is the second leading cause of ED visits resulting in hospitalization among patients aged 65 and older (5.4% of encounters for those over 65), with cardiac arrhythmias ranking fifth among causes of ED visits resulting in hospitalization within this age group (4.3% of encounters for those over 65) [6].

A large proportion of patients who present to the ED with cardiac-related complaints can be discharged from the ED with appropriate outpatient follow-up. Various clinically validated tools have been created to aid in the risk stratification of patients presenting to the ED and can help identify those at low-risk of near-term events who may be safely discharged to home with close follow-up. A recent clinical trial supported the concept of triaging low-risk chest pain patients via the HEART Pathway to early discharge from the ED and demonstrated no significant increase in major adverse cardiac events within 30 days [7]. The Emergency Heart Failure Mortality Risk Grade (EHMRG) is a similar tool that has been developed for patients presenting to the ED with CHF [8]. Unfortunately, inadequate care coordination of patients that are discharged from the ED lessens much of the benefit to both patients and the healthcare system that can be derived from these risk stratification and early discharge strategies. This failure to ensure timely follow-up is a source of potentially avoidable ED revisits and their associated healthcare costs, including preventable admissions [9]. Recent data spanning over 50 million ED encounters from the multistate Healthcare Cost and Utilization Project indicate that within 30 days of an index ED visit, the overall rate of return for any cause was 19.9% [10]. Approximately 30% of patients revisiting the ED are admitted [9]. Although there is a growing literature base demonstrating the benefits of improved care coordination among patients discharged from the hospital following an admission [11–14], there remains a paucity of data specifically examining the impact of improved care coordination among patients being discharged directly from the ED.

In order to improve care-coordination following an ED visit among cardiac patients, we developed HEART TRACKS—a structured transitional care pathway for patients presenting with cardiac-related complaints. This program was developed collaboratively between the Cardiology and Emergency Departments at SoutheastHEALTH, with active engagement of senior physician and nursing leaders from both departments as well as executive leaders from Quality Management and Process Improvement.

### Goals of this investigation

The purpose of this study was to assess the impact of HEART TRACKS on ED discharges, cardiac-related and all-cause 30-day revisits and admissions, and institutional revenues.

## Methods

### Study design

This was a single-center prospective observational cohort study of patients enrolled in the HEART TRACKS transitional care pathway.

### Setting

The study was conducted in a general, full-service, acute-care hospital in Southeast Missouri with an annual ED volume of approximately 35,000 patients. The acute-care hospital includes an integrated cardiovascular service line offering a full range of general and interventional cardiology services as well as clinical cardiac electrophysiology and cardiothoracic surgery. Prospective data on enrolled patients were collected from the beginning of April 2016 through the end of April 2017; historical data from the beginning of April 2015 through the end of March 2016 was used for baseline comparisons.

### Participants

All patients aged 18-years and older presenting with a cardiac-related complaint (e.g., chest pain, CHF, or an arrhythmia) and who were discharged from the emergency department were considered eligible for enrollment into HEART TRACKS.

### Transitional care pathway design

**Fig. 1:**
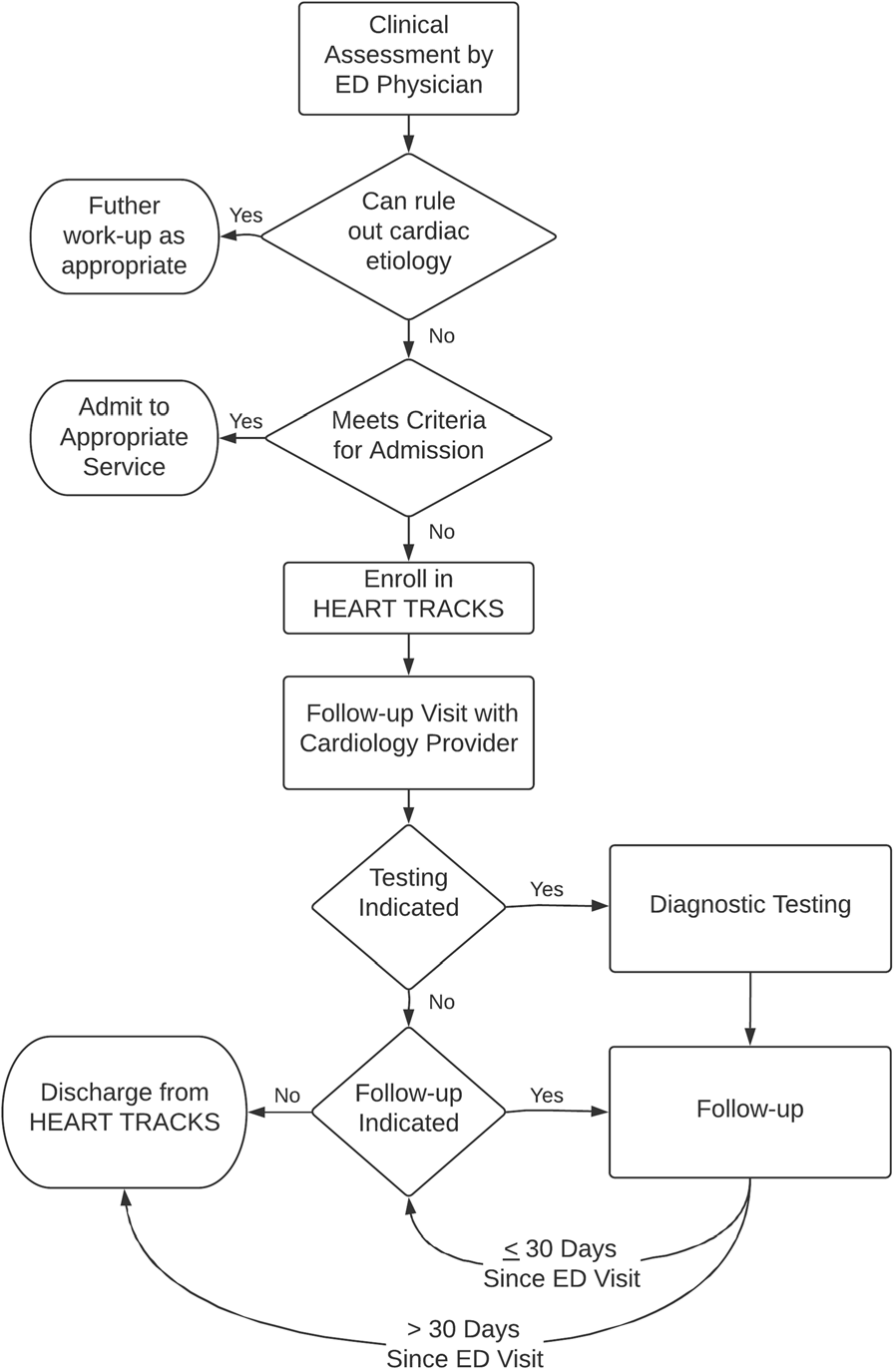
Patient flow through the ED and HEART TRACKS. After assessment by an ED physician, patients deemed to have a confirmed or suspected cardiac-related condition and who were considered to be at low-risk for discharge were enrolled in HEART TRACKS. Enrolled patients were subsequently contacted and scheduled for follow-up with a cardiology provider, and appropriate testing and follow-up was performed. Upon completion of testing and immediate follow-up, patients were discharged from HEART TRACKS

Structured care pathways for patients presenting to the ED with chest pain, heart failure, or an arrhythmia were developed as discussed below to help ED providers identify low-risk patients eligible for early discharge from the ED. The only general exclusions to the application of these pathways were age < 18-years, life expectancy < 1 year and/or patients opting for hospice care, or a noncardiac medical, surgical, or psychiatric illness determined by the ED provider to require admission. Those patients selected for early discharge and who—at the discretion of the ED provider—were deemed to require further cardiology follow-up or evaluation were enrolled into HEART TRACKS at the time of ED discharge. Enrolled patients were subsequently scheduled to see a cardiology provider within an integrated cardiovascular outpatient clinic of advanced practice nurses (APRN’s) and physicians. Coordination of follow up visits was performed by a registered nurse (HEART TRACKS Coordinator) within the clinic. Figure 1 summarizes the flow of patients through the ED and into the HEART TRACKS transitional care pathway. ED providers were able to enroll patients in HEART TRACKS 24-h a day, 7-days a week. Enrollment requests submitted on weekends and holidays were not processed by the HEART TRACKS coordinator until the next business day.

### Interventions

#### Patient risk stratification

For chest pain patients, a care pathway centered around the HEART score [15–19] was implemented as described previously [7]. A HEART score ≤ 3 was considered low risk with patients eligible for early discharge, and scores ≥4 were considered high risk with a recommendation for cardiology consultation in the ED or admission; patients with new ST-segment elevations ≥ 1 mm were excluded. For heart failure patients, a care pathway centered around the EHMRG [20] was implemented as described previously [8]. Predicted 7-day mortality rates of 0.3% and 0.7% were considered low risk with patients eligible for early discharge, 2% was considered intermediate risk with patients eligible for discharge if clinical improvement was seen during their ED stay, and 3.5% and 8.2% were considered high risk with a recommendation for cardiology consultation in the ED or admission. For arrhythmia patients, those with stable or successfully treated supraventricular tachycardia, or atrial fibrillation or flutter without high risk-features (i.e., poor rate control, hypotension, decompensated CHF, or CHA_2_DS_2_-VASc Score [21] ≥ 3 and not on systemic anticoagulation) were considered low risk and eligible for early discharge; all others, including those patients with sustained ventricular tachycardia, were deemed high risk with a recommendation for cardiology consultation or admission. As part of the chest pain pathway, the HEART score was automatically calculated for all patients presenting with chest pain and made available to ED providers at the time of the patient encounters; the use of serial Troponin testing was at the discretion of the ED providers, and typically depended upon the elapsed time between reported symptoms and presentation to the ED. For the CHF pathway, a web-based tool for calculating the EHMRG was made available to providers, with data entry and score calculations performed by the ED nursing staff on all patients presenting with CHF [22]. Use of these pathways were strongly encouraged, but were not mandated, with the ultimate decisions regarding admission or early discharge being left to the discretion of the ED providers.

### HEART TRACKS enrollment and follow-up

Enrollment into HEART TRACKS was initiated by a single electronic order placed into the EHR system by the ED provider, which resulted in an electronic notification containing the patient’s demographics, primary diagnosis, date of service, and contact information being sent to the HEART TRACKS Coordinator. Enrolled patients received specific teaching and instructions at the time of discharge from the ED regarding their enrollment and plans for further cardiology evaluation post discharge. The coordinator reviewed all incoming enrollment requests and associated ED documentation for patient encounters and scheduled timely follow-up with a cardiology APRN or cardiologist at their discretion. Scheduling decisions were based on a number of factors, including, but not limited to, acuity, provider availability, patient proximity to the practice’s primary clinic or outreach office location, anticipated need for invasive versus non-invasive testing, and patient preferences. Additional cardiac testing was ordered at the time of a provider encounter as appropriate. Once such testing was completed and the results reviewed with the patient, further cardiology follow-up was scheduled if needed; otherwise, the patient was discharged from HEART TRACKS, at which time their primary care provider (PCP) was notified by correspondence of the patient’s ED visit. All ED and cardiology provider encounter notes, as well as test results, were made available to the PCP via the organization’s electronic health information exchange portal. A custom HEART TRACKS Management Tool was created using a structured spreadsheet (Excel, Microsoft Corporation, Redmond, WA) to facilitate the tracking of patient enrollment, follow-up scheduling and testing, and outcomes. The Management Tool captured basic demographic information and included a built-in dashboard that provided instantaneous access to metrics such as enrollment volumes, mean times to post-ED contact and post-ED visit, patient follow-up rates, and 30-day patient outcomes. Data entry into the Management Tool was primarily performed by the HEART TRACKS Coordinator.

### Data sources and collection

Data regarding ED visits and hospitalizations were obtained from both the ED and hospital’s EHR systems (Optum ED Pulse Check, Optum Clinical Solutions, Inc., Eden Prairie, MN; Soarian Clinicals, Cerner Corporation, Kansas City, MO). Data regarding cardiology provider visits and testing were obtained from the ambulatory EHR system (NexGen Ambulatory EHR, NexGen Healthcare, Horsham, PA). A systematic search of ED provider documentation for the period spanning April 2015 through April 2017 was performed by identifying patients who had been triaged with a tentative cardiac-related presenting problem (selected by an ED nurse from a standardized list) or had been referred to cardiology. The search was then further refined by manually reviewing and generating a list of 551 unique provider-entered (free-text) final diagnoses that were classified as being cardiac-related. This list of final diagnoses was used in subsequent queries to identify those patients having presented with cardiac-related complaints during the pre- and post-HEART TRACKS launch periods, regardless of what was recorded as their presenting problem upon initial triage. Although consideration was given to using coder-assigned International Classification of Diseases (ICD) diagnostic codes instead of provider-entered final diagnoses, the former was felt to be less reliable for identifying patients with cardiac-related complaints consistently during the pre-HEART TRACKS period due to the transition from ICD-9 to ICD-10 in October 2015, along with changes in coding specificity accompanying the change which may have affected how codes were assigned before versus after the transition.

### Primary data analysis

For statistical analyses, we used Microsoft Excel (Microsoft Corporation, Redmond, WA) and MedCalc (MedCalc Software, Seoul, Republic of Korea). Statistical inference was performed using two-sample *t*-tests and χ^2^ tests for continuous and categorical data, respectively; when small categorical sample sizes were examined (*n* ≤ 5), Fisher’s exact test was used. For comparisons of ordinal or continuous data that was non-Normally distributed, the Mann-Whitney U test was used. *p* < .05 (i.e., α = 0.05) was considered significant unless otherwise noted. We calculated 95% binomial exact confidence intervals (CIs) where reported. Statistical control charts were created using Excel templates made available by the American Society for Quality (http://asq.org).

### Cost analysis

Implementation costs considered included administrative and provider costs associated with seeing patients in outpatient follow-up. The fixed salary of the HEART TRACKS coordinator was allocated to administrative costs. A time-driven, activity-based costing model was used to calculate expenses for cardiology staffing requirements from a pool of eight cardiologists, two cardiology APRNs, ten RN/LPNs, and five patient care technicians. Provider costs, *C*_*Prov*_, were calculated as the sum of each provider’s annual salary allocated to ambulatory care provision, *S*_*i*_, weighted by the proportion of HEART TRACKS patients seen:

$$ {C}_{Prov}={\varSigma}_i\left({S}_i\cdotp {N}_{i\mathrm{HT}}/{N}_{i\mathrm{T}}\right) $$

where N_*i*HT_ is the number of HEART TRACKS patients seen by a specified provider *i* and N_*i*T_ is the total annual capacity of ambulatory patients for that provider.

No direct IT costs were incurred, as calculation of the HEART Score was performed through a preexisting (although previously unutilized) feature of the ED’s EHR, and access to the EHMRG was made via the simple addition of a web link to the clinical providers’ workflow in the EHR. ED provider orders for enrollment into HEART TRACKS and communication of patient information to the HEART TRACKS Coordinator utilized pre-existing functionality within the Hospital’s clinical information systems and required minimal effort to implement and test. The HEART TRACKS Management Tool was created by the authors.

Positive revenue contributions, R_Pos_, from outpatient visits and ancillary testing were estimated from the mean Medicare reimbursement rates for provider visits as well as from ancillary testing services performed:

$$ {\mathrm{R}}_{\mathrm{Pos}}={\mathrm{N}}_{\mathrm{HTv}}\cdotp {\mathrm{C}}_{\mathrm{v}}+{\Sigma}_{s,t}\ \left(s\cdotp {\mathrm{C}}_t\right) $$

where N_HTv_ is the number of HEART TRACKS patient visits, C_v_ is the mean reimbursement for a provider visit (CPT 99201–99205), and C_*t*_ is the reimbursement rate for an ancillary test *t* performed on *s* number of patients (the observed frequency of testing performed among HEART TRACKS patients is given in Table 1); revenues from downstream procedures such as diagnostic cardiac catheterization and other invasive studies were not included.

**Table 1:** HEART TRACKS enrollment and provider visit data

	N
Total number of patients enrolled in HEART TRACKS	572
Mean time (range) to post-ED visit contact	1.4 (0–17) Days
Mean time (range) to scheduled appointment with cardiology provider	7.0 (0–44) Days
Patients seen by cardiology provider within 7 days of ED visit	220
Patients seen by cardiology provider within 8–14 days of ED visit	58
Patients seen by cardiology provider over 15 days after ED visit	28
Patients keeping follow-up visit appointment	306
Appointment with APRN only	191
Appointment with MD ± APRN	110
Appointment for testing only (as ordered from ED)	5
Patients new to cardiology practice (not seen within 3 years)	220
Patients undergoing ancillary testing	191^a^
SPECT stress test	65
Stress echocardiogram	21
Plain treadmill stress test	41
Echocardiogram	61
14- to 30-day event monitor	21
24- or 48-h Holter monitor	8
Other	45

^a^The total number of tests exceeds 191 as some patients underwent multiple tests

Negative revenue contributions, R_Neg_, were estimated from projected revenue declines associated with a higher rate of initial discharges from the ED resulting in fewer primary hospital admissions, adjusted by gains/losses associated with changes in ED revisit volumes and secondary hospital admissions:

$$ {\mathrm{R}}_{\mathrm{Neg}}={\mathrm{R}}_{\mathrm{PAdm}}+{\mathrm{S}}_{\mathrm{R}\mathrm{ev}+\mathrm{SAdm}} $$

where R_PAdm_ is the loss of revenue from reduced primary admissions, and S_Rev + SAdm_ is a revenue adjustment accounting for changes in ED revisit volumes and secondary hospital admissions.

R_PAdm_ and S_Rev + SAdm_ were estimated as follows:

$$ {\mathrm{R}}_{\mathrm{P}\mathrm{Adm}}={\mathrm{N}}_{\mathrm{CR}}\cdotp \left({\mathrm{D}}_{\mathrm{P}}-{\mathrm{D}}_{\mathrm{B}}\right)\cdotp \left({\mathrm{P}}_{\mathrm{ADM}}-{\mathrm{P}}_{\mathrm{ED}}\right) $$

$$ {\mathrm{S}}_{\mathrm{R}\mathrm{ev}+\mathrm{SAdm}}={\mathrm{N}}_{\mathrm{CR}}\cdotp \left[{\mathrm{D}}_{\mathrm{B}}\cdotp {\mathrm{R}}_{\mathrm{B}}\cdotp \left({\mathrm{P}}_{\mathrm{ED}}+{\mathrm{A}}_{\mathrm{B}}\cdotp \left({\mathrm{P}}_{\mathrm{A}\mathrm{DM}}-{\mathrm{P}}_{\mathrm{ED}}\right)\right)-{\mathrm{D}}_{\mathrm{P}}\cdotp {\mathrm{R}}_{\mathrm{P}}\cdotp \left({\mathrm{P}}_{\mathrm{ED}}+{\mathrm{A}}_{\mathrm{P}}\cdotp \left({\mathrm{P}}_{\mathrm{A}\mathrm{DM}}-{\mathrm{P}}_{\mathrm{ED}}\right)\right)\right] $$

where N_CR_ is the annual number of patients presenting to the ED with a primary cardiac-related complaint, D_B_ and D_P_ are the observed baseline and post-implementation discharge rates from the ED, respectively; R_B_ and A_B_ are the baseline all-cause revisit and secondary hospital admission rates, respectively; R_P_ and A_P_ are the post-implementation all-cause revisit and secondary hospital admission rates, respectively; and P_ED_ and P_ADM_ are the average hospital Medicare payment rates for a cardiac-related ED visit (CPT 99285) or observation admission (C-APC 8011), respectively.

## Results

### HEART TRACKS enrollment

Enrollment and provider visit information is summarized in Table 1. In the first 12-months after HEART TRACKS implementation, 2049 (5.9%) out of a total of 34,526 ED patients presented with a primary cardiac-related complaint; of these, 1161 patients (57%) were discharged from the ED, with 572 patients (49%) enrolled into HEART TRACKS.

Chest pain was the leading complaint (85%) among patients presenting to the ED with a primary cardiac-related complaint who were enrolled in HEART TRACKS. Women represented just over half of enrollees, while minorities represented just under 15%. The median time to first patient contact by the HEART TRACKS coordinator following an ED visit was 1 day (range 0–17 days), with the first contact being made within 72 h of the index ED visit in 99% of the patients who kept their follow-up appointment. The median time for an appointment with a cardiology provider was 5 days (range 0–44 days); over 90% of follow-up visits occurred within 14 days of the index ED visit.

The overall rate of follow-up was 53% among enrolled patients, with just over 70% of those followed up being new to the cardiology practice (defined as not having been seen within three years). About 62% of patients had a visit with a cardiology APRN only, while 36% were seen by a physician; the remainder presented for follow-up testing only as ordered from the ED (i.e., no visit scheduled with a provider; results forwarded to primary care provider). Approximately 62% of patients evaluated by a provider underwent some form of ancillary testing; the rate of testing among all enrolled patients was 33%, which is comparable to rates of ambulatory cardiac testing reported elsewhere [7]. Among the 220 patients who were new to the cardiology practice, 84 (38%) were found to have conditions that warranted ongoing cardiology follow-up after discharge from HEART TRACKS. Among the 266 patients enrolled at the time of ED discharge who did not follow-up with a cardiology provider, 254 (95%) either could not be reached by the HEART TRACKS Coordinator for scheduling of an appointment with a provider or declined follow-up and 12 (5%) failed to keep their scheduled appointment with a provider.

### ED discharge rates, 30-day returns and admissions

**Fig. 2:**
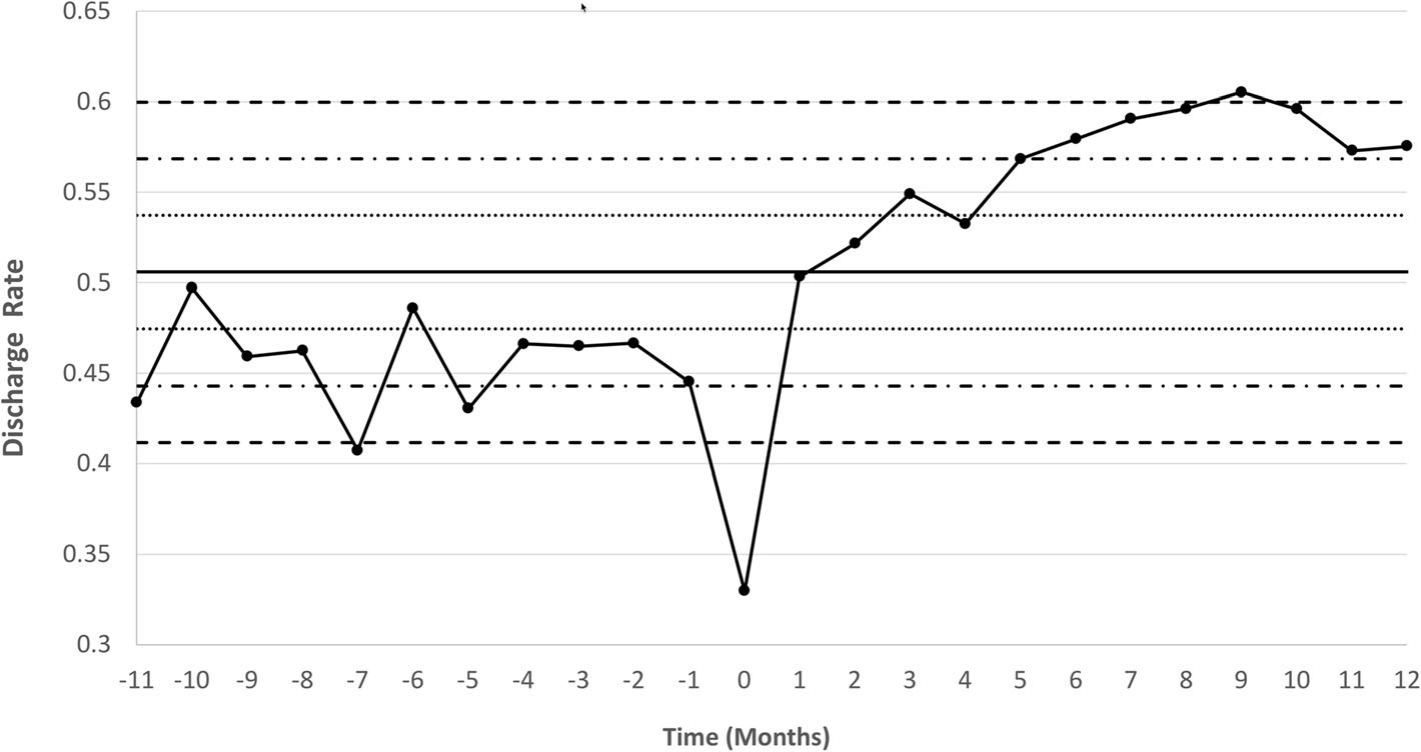
Statistical process control chart showing monthly ED discharge rate (i.e., number of patients discharged from the ED divided by the total number of patients who presented to the ED) among those patients presenting with a primary cardiac related-complaint. Time point 0 represents the launch date of HEART TRACKS, with time point 1 being the first full month post-launch. The solid line represents the mean the discharge rate. Dotted lines represent one standard deviation away from the mean, mixed dotted-dashed lines represent two standard deviations away from the mean, and full dashed lines represent three standard deviations away from the mean

Data on ED discharges and 30-day returns are summarized in Table 2. In the 12-month period following the launch of HEART TRACKS, the discharge rate from the ED for patients presenting with a primary cardiac-related complaint increased from 44.4% in the preceding 12-months to 56.6% (∆ 12.2%, 95% CI 9.0 to 15%, *P* < 0.0001). Figure 2 shows the statistical process control plot for the monthly discharge rate of patients presenting to the ED with a primary cardiac-related complaint, demonstrating an immediate and sustained increase in the rate of discharges from the ED following HEART TRACKS implementation. A sharp decline in ED discharges in the month immediately prior to the launch of HEART TRACKS was observed and coincided with pre-launch ED provider education regarding the use of the HEART score and EHMRG risk grade for patient risk stratification: the failure to make these tools available to providers until launch day may have led to a reluctance towards discharging patients without documentation of low-risk status. During this same period, there were nonsignificant declines in the rates of cardiac-related (12.0 versus 10.7%, ∆ -1.3%, 95% CI -4.2 to 1.6%, *p* = 0.3696) and all-cause (37.3 versus 34.0%, ∆ -3.3%, 95% CI –7.6 to 1.0%, *p* = 0.131) 30-day revisits; however, there was a significant drop in the rate of admission at second ED visit (45.6 versus 37.7%, ∆ -7.9%, 95% CI -15 to − 0.40%, *p* = 0.0338).

**Table 2:** ED discharges and 30-day outcomes among patients with cardiac-related complaints

	12-Months Pre-HEART TRACKS Launch (*N* = 1920)	12-Months Post-HEART TRACKS Launch (*N* = 2049)
Discharged to Home from ED	853	1161
Enrolled in HEART TRACKS		572
Not enrolled in HEART TRACKS		589
Cardiac-Related Revisits to ED	102	124
Enrolled in HEART TRACKS		22
Not enrolled in HEART TRACKS		102
All-Cause Revisits to ED	318	395
Enrolled in HEART TRACKS		89
Not enrolled in HEART TRACKS		306
Admitted at Second ED-Revisit	145	149
Enrolled in HEART TRACKS		21
Not enrolled in HEART TRACKS		128

Among all patients presenting with a primary cardiac-related complaint that were discharged from the ED following HEART TRACKS implementation, those enrolled in HEARTS TRACKS had significantly lower risks of cardiac-related ED revisits (RR 0.22, 95% CI 0.14 to 0.35, *P* < 0.0001), all-cause ED revisits (RR 0.30, 95% CI 0.24 to 0.37, P < 0.0001), and admission at second ED visit (RR 0.56, 95% CI 0.38 to 0.83, *p* = 0.0047) compared to those patients who were not enrolled in HEART TRACKS. Only 2 of the 254 enrolled patients who could not be reached or declined follow-up returned within 30 days with a cardiac-related complaint.

The cardiac-related reasons for ED returns among HEART TRACKS enrolled patients were: chest pain (16 patients, including one ST-elevation myocardial infarction whose calculated HEART score was 3 at the time of their index ED visit 7-days prior, as well as one non-ST-elevation myocardial infarction), CHF (3 patients, including one end-stage heart failure presenting with asystolic arrest), and arrhythmia (3 patients); 8 of the 19 patients had seen a cardiology provider prior to their ED revisit. An additional 30 patients returned to the ED within 30 days for non-cardiac-related reasons, with musculoskeletal and gastrointestinal complaints accounting for 50% of these. The 30-day rate of return for myocardial infarction among enrolled patients was 0.35%, comparable to previously reported MACE rates among patients with HEART scores ≤ 3 [32].

Among the 589 non-enrolled patients who were discharged from the ED after having presented with a primary cardiac-related complaint, there was a subgroup of 33 patients who were referred to cardiology for follow-up outside of the HEART TRACKS pathway (19 of these patients were new patients to the cardiology practice). Patients in this subgroup had a significantly increased risk of a cardiac-related return to the ED as compared to their enrolled counterparts (RR 3.9, 95% CI 1.6 to 9.7, *p* = 0.003), with 5 (15%) returning to the ED with a cardiac-related complaint within 30 days. Notably, three of these returns occurred within 72-h of the index ED visit. Compared to patients who had been enrolled in HEART TRACKS, the median time to first patient contact following the ED visit was significantly prolonged to 3 days (*P* = 0.0164); however, the median time for an appointment with a cardiology provider was unchanged at 5 days.

### Disparities in care delivery

In the first 120 days following program implementation, a significant gender disparity was noted in the rates of ancillary cardiac testing, with men undergo testing at a rate 1.5× that of women (44.3 versus 28.6%, ∆ 15.7%, 95% CI 1.0 to 29.4%, *P* = 0.0372); this was associated with a 2.5× increase in the 30-day all-cause revisit rate among women as compared to men (16.9 versus 6.8%, ∆ 10.1%, 95% CI 0.2 to 20.6%, *P* = 0.0440).

Interviews with ED and Cardiology providers and review of clinical documentation suggested that a significant factor contributing to this disparity was a subset of ED physicians who were advising patients at the time of their ED visit that they would be scheduled for further cardiac testing through HEART TRACKS. Cardiology APRNs reported that male patients were generally more insistent that such testing be performed at the time of their follow-up visit as compared to female patients, even if testing was not indicated according to current clinical practice guidelines and Appropriate Use Criteria [23]. It was hypothesized that the lower rate of testing performed among women after the expectation had been set in the ED for the need to perform such testing was contributing to their higher revisit rate.

These findings were shared with all ED and Cardiology providers at the end of the initial 120 days post HEART TRACKS launch, resulting in the following interventions: (1) scripted nursing-driven patient education regarding the HEART TRACKS program was incorporated into the discharge process; (2) ED providers were asked to defer all discussions regarding the need for follow-up testing to Cardiology so as to ensure that any testing performed was in accordance with current Appropriate Use Criteria and clinical practice guidelines. In the immediate 120 days following this intervention, the observed disparity in the rates of ancillary cardiac testing among men and women was eliminated (31.6 versus 32.4%, ∆ 0.7%, 95% CI -12.9 to 13.9%, *P* = 0.9160), as was the disparity in 30-day all-cause revisit rates among women as compared to men (10.5 versus 11.4%, ∆ 0.9%, 95% CI -8.1 to 10.9%, *P* = 0.8437).

### Cost analysis

Institutional costs associated with administrative overhead and provider staffing totaled $63,590. This included provider staffing expenses (physician, APRN, and supporting clinical staff salaries) of $52,510 associated with seeing those patients who kept their follow-up appointments, as well as a $13,080 salary component for the HEART TRACKS coordinator, who was considered a 0.25 FTE at the average weekly hourly commitment to HEART TRACKS required for the number of patients enrolled in this study.

Positive institutional revenue contributions from provider visits and ancillary outpatient testing totaled an estimated $135,820. Negative institutional revenue contributions due to reduced rates of hospitalizations totaled an estimated $265,080; this was offset by a small gain in revenue of $21,480 from a numerically higher number of ED return visits and associated secondary hospitalizations. The net institutional financial impact—accounting for program costs and the explicit revenue gain/losses cited above—was a loss of $171,370 in the 12-months following HEART TRACKS implementation, or approximately $300 per enrolled patient.

## Discussion

### Impact of transitional care pathway implementation

#### Impact on ED discharges and 30-day outcomes

Implementation of the HEART TRACKS transitional care pathway resulted in a significant increase in the rate of ED discharges among patients presenting with cardiac-related complaints (for both primary visits and revisits), without adversely affecting 30-day cardiac-related and all-cause revisits rates or leading to significant adverse patient outcomes; indeed, the low 30-day acute myocardial infarction rate supports the use of the HEART score for identifying low-risk chest pain patients eligible for early discharge from the ED. ED discharge rates increased steadily and remained elevated after the launch of HEART TRACKS, suggesting that implementation of a transitional care pathway can be a sustainable and durable change to patient care workflow.

Although enrollment into HEART TRACKS was associated with a significantly lower risk of ED revisits, the absolute rate of revisits did not significantly change in the 12-months following program launch. This apparent paradox is explained by significantly higher cardiac-related (17.3 versus 12.0%, ∆ 5.3%, 95% CI 1.6 to 9.3%, *P* = 0.0041) and all-cause (52.0 versus 37.3%, ∆ 14.7%, 95% CI 9.3 to 19.9%, *P* < 0.0001) 30-day revisit rates among non-enrolled patients compared to patients in the preceding 12-months. This suggests that among the pool of patients presenting with cardiac-related complaints, those who were at low-risk for revisits were selectively enrolled into HEART TRACKS. It is unclear what factors led to providers’ selective enrollment of patients being discharged. It may be that providers had a lower clinical suspicion for a cardiac etiology for symptoms among non-enrolled patients; nevertheless, given their high revisit rate, future efforts targeting this group may prove effective in lowering absolute revisit rates. Although a significant number of enrolled patients either declined follow-up or could not be reached, the return rates were so low in this subgroup that there would likely be little benefit from efforts aimed at improving rates of follow-up. It is noteworthy that patients referred for cardiology follow-up outside of the HEART TRACKS pathway had a significantly elevated risk of a cardiac-related return, suggesting that a clear follow-up plan and minimizing time to first patient contact post-discharge can play important roles in reducing ED revisits.

#### Impact on institutional revenues

Costs of program implementation were predominantly driven by direct provider and ancillary clinical staff salaries, as well as by having a program coordinator to facilitate the timely scheduling and evaluation of patients. These costs were largely offset by direct revenue gains generated from provider visits and ancillary testing services; however, the transitional care pathway’s effect of shifting patient evaluations away from hospital-based and towards ambulatory settings through increased ED discharges had a significant net negative impact on institutional revenues in the current fee-for-service model. Such losses may be accentuated at smaller institutions not offering ambulatory services, or when dealing with patient populations where there is minimal revenue to be derived from ancillary testing.

#### Disparities in care delivery

Prior studies have identified gender disparities in cardiovascular care access and delivery, although the causes are not well understood [24–31]. In the current study, we identified an early gender disparity in the rates of cardiac testing which appeared to impact 30-day revisit rates. Although a definitive cause cannot be established, qualitative feedback from providers and a targeted intervention that eliminated the observed disparity suggests that it was mediated by provider-introduced biases in the ED that set patients’ expectations and impacted subsequent discussions and medical decision making with specialists. This would underscore the importance of consistent messaging to patients—especially when transitions of care occur across specialties—in order to facilitate the delivery of guideline-directed testing and therapies.

### Limitations

Several limitations of this study should be noted. First, this was a single-center study with data on return ED visits and admissions being limited to patients presenting back to the same hospital; accordingly, the *absolute* revisit rates (including possible revisits to outside institutions) as reported are underestimated by an unknown amount, although the *relative* revisit rates between enrolled and non-enrolled patients and between the pre- and post-HEART TRACKS launch periods are likely minimally impacted. Similarly, the reported 30-day myocardial infarction rate is limited to returns to the same hospital, and thus may underestimate the actual absolute event rate.

Another limitation is that enrollment of patients into HEART TRACKS was not randomized, thereby introducing potential biases into the patient selection process; furthermore, no specific data was collected as to the reasons why ED providers elected not to enroll otherwise eligible patients into the program, nor was any data collected as to the frequency of serial troponin testing or its impact on clinical decision making. It is possible that many of the patients who were not enrolled in HEART TRACKS were felt by the ED providers to have a low likelihood of having a cardiac etiology for their symptoms, as suggested by the observation that the rate of non-cardiac-related revisits in non-enrolled patients (34.6%) was significantly higher than the baseline rate of non-cardiac-related revisits (25.3%) seen in the preceding 12-months (∆ 9.3%, 95% CI 4.4 to 14.2%, *P* = 0.0001). Furthermore, the overall rate of follow-up was only 53% among enrolled patients, with the majority of those who did not follow-up either declining an appointment with a cardiology provider or being unreachable, arguing that there is room for optimization of the patient selection and enrollment process. Unfortunately, the design and implementation of this study did not include patient feedback, precluding identification of consumer-side factors which may have impacted follow-up.

Finally, the financial impact analysis is limited by the use of institution-specific provider costs and revenue estimates utilizing single payor (i.e.*,* Medicare) rates, and the inability to account for downstream revenue gains and losses. A comprehensive financial impact analysis would require longitudinal cost data at the institutional and payor levels for the entire cohort of ED patients presenting with a primary cardiac-related complaint and is beyond the scope of this study. Nonetheless, the analysis presented herein suggests that the observed increase in ED discharge rates results in a decline in net institutional revenue due to the shifting of patient care towards the lower cost ambulatory setting.

## Conclusion

In summary, a transitional care pathway offering prompt follow-up for patients being discharged from the ED who present with a primary cardiac-related complaint can significantly enhance the rate of discharges from the ED without adversely affecting patient outcomes. The shifting of cardiac evaluations from hospital-based to ambulatory care settings may lead to significant cost-savings for payors and improve the value of healthcare delivery. Further work will be required to determine if such transitional care pathways can be extended to other patient populations and if shared savings can be achieved between providers and payors through alternate payment models.

## Abbreviations

ED: Emergency department
EHMRG: Emergency heart failure mortality risk grade
